# Expression Profiles of Kidney Mitochondrial Proteome during the Progression of the Unilateral Ureteral Obstruction: Focus on Energy Metabolism Adaptions

**DOI:** 10.3390/metabo12100936

**Published:** 2022-10-02

**Authors:** Ariadna Jazmín Ortega-Lozano, Alexis Paulina Jiménez-Uribe, Ana Karina Aranda-Rivera, Leopoldo Gómez-Caudillo, Emmanuel Ríos-Castro, Edilia Tapia, Belen Bellido, Omar Emiliano Aparicio-Trejo, Laura Gabriela Sánchez-Lozada, José Pedraza-Chaverri

**Affiliations:** 1 Department of Biology, Faculty of Chemistry, National Autonomous University of Mexico (UNAM), Mexico City 04510, Mexico; 2 Genomic, Proteomic, and Metabolomic Unit (UGPM), LaNSE, Cinvestav-IPN, Mexico City 07360, Mexico; 3 Department of Cardio-Renal Physiopathology, National Institute of Cardiology “Ignacio Chávez”, Mexico City 14080, Mexico

**Keywords:** unilateral ureteral obstruction (UUO), kidney fibrosis, mitochondria proteome, energy metabolism

## Abstract

Kidney diseases encompass many pathologies, including obstructive nephropathy (ON), a common clinical condition caused by different etiologies such as urolithiasis, prostatic hyperplasia in males, tumors, congenital stenosis, and others. Unilateral ureteral obstruction (UUO) in rodents is an experimental model widely used to explore the pathophysiology of ON, replicating vascular alterations, tubular atrophy, inflammation, and fibrosis development. In addition, due to the kidney’s high energetic demand, mitochondrial function has gained great attention, as morphological and functional alterations have been demonstrated in kidney diseases. Here we explore the kidney mitochondrial proteome differences during a time course of 7, 14, and 21 days after the UUO in rats, revealing changes in proteins involved in three main metabolic pathways, oxidative phosphorylation (OXPHOS), the tricarboxylic acid cycle (TCA), and the fatty acid (FA) metabolism, all of them related to bioenergetics. Our results provide new insight into the mechanisms involved in metabolic adaptations triggered by the alterations in kidney mitochondrial proteome during the ON.

## 1. Introduction

Obstructive uropathy is a pathology characterized by the disruption of the normal urine flow caused by a great diversity of etiologies, such as urolithiasis, prostatic hyperplasia in males, tumors, congenital stenosis, and others. If the obstruction causes irreversible damage to the kidney, it is referred as obstructive nephropathy (ON). Some of the most noticeable events during the ON pathophysiology include increased intratubular pressure, an acute increase of renal blood flow (RBF) followed by its decrease, and the consequent reduction of glomerular filtration rate (GFR). As the obstruction persists, tubular acidification, apoptosis of epithelial cells, tubular atrophy, inflammation, fibrosis, and finally, renal failure occurs [1,2,3,4].

Unilateral ureteral obstruction (UUO) in rodents is an experimental model extensively used to explore the mechanisms and pathways involved in ON development. It replicates alterations in RBF and GFR, interstitial inflammation, tubular dilation, tubular atrophy, and the progressive development of fibrosis observed in clinics [5,6,7,8]. Moreover, mitochondrial–dependent oxidative stress and apoptosis have also been observed during the UUO [9,10].

Since kidneys are highly energy–demanding organs for their filtration and reabsorption functions, mitochondria, as the bioenergetics centers of the cells, are key organelles for their proper functions, particularly for tubular segments [11]. In fact, in different models of kidney damage, mitochondrial structural and functional alterations have been described, such as swelling, loss of cristae, and a decrease in the activity of the electron transport system (ETS), which triggers redox imbalance and mitochondrial–dependent apoptosis [12,13,14,15,16,17,18].

Recently, our group reported that kidney mitochondrial biogenesis is decreased along with the progression of UUO–induced kidney fibrosis in rats, resulting in lower mitochondrial mass compared to the sham–operated group. Furthermore, mitochondrial function is compromised since oxidative phosphorylation (OXPHOS) is decreased, possibly due to alterations in complexes I or II of the ETS [12].

In addition, other reports indicate that after one week of UUO in mice, there are energetic adaptations associated with increased glycolytic metabolism, which promotes the development of fibrosis [19,20]. This could point to the fact that mitochondrial dysfunction triggers the metabolic reprogramming of kidney cells to cope with the pathological changes in the UUO.

The mitochondrial proteome comprises around 1300 nuclear and mitochondrial DNA–encoded proteins required for the proper functioning of several pathways performed by this organelle, such as OXPHOS, tricarboxylic acid cycle (TCA), fatty acid (FA) β–oxidation, amino acids and pyrimidine biosynthesis, calcium homeostasis, apoptosis regulation, redox balance, among others [21,22].

Given that mitochondria are key organelles for kidney function where several metabolic processes are carried out, we consider that exploring the mitochondrial proteome could be very useful to have a broader view of the changes that occur in these processes during the UUO, as well to elucidate their participation in the ON pathophysiology.

In the present work, using liquid chromatography–tandem mass spectrometry (LC–MS/MS), we performed an exploratory analysis of the kidney mitochondrial proteome of obstructed kidneys during a time course of 7, 14, and 21 days after UUO in rats.

Our results reveal evident changes in proteins involved in three main pathways of mitochondrial bioenergetics, namely OXPHOS, TCA, and FA metabolism. Moreover, these changes suggest that alterations in the mitochondrial proteins involved in the mentioned metabolic processes could drive energy metabolism reprogramming in renal cells that could favor the pathological process.

## 2. Materials and Methods

### 2.1. Reagents

Acrylamide, ammonium persulfate (APS), bromophenol blue, fat–free bovine serum albumin (BSA), D–mannitol, 4 – (2–hydroxyethyl) – 1–piperazineethanesulfonic acid (HEPES), sodium succinate dibasic, sodium phosphate dibasic, sodium phosphate monobasic (NaH_2_PO_4_), sodium dodecyl sulfate (SDS), sucrose, 1,2–Bis (dimethylamino) ethane (TEMED), glycerol, glycine, phenylmethylsulfonyl fluoride (PMSF), sodium deoxycholate, sodium fluoride (NaF), sodium orthovanadate (Na_3_VO_4_), sodium phosphate dibasic (Na_2_HPO_4_), sodium pyrophosphate (Na_4_P_2_O_7_), tris–base, Tween–20, urea, and β–mercaptoethanol were purchased from Sigma–Aldrich (St. Louis, MO, USA). Ethanol, ethylenediaminetetraacetic acid disodium salt dihydrate (EDTA), isopropanol, methanol, and sodium chloride (NaCl) were purchased from JT Baker (Xalostoc, Edo. Mexico City, Mexico). Sodium pentobarbital (Sedalphorte^®^) was purchased from Salud y Bienestar Animal S.A. de C.V. (Mexico City, Mexico). The protease inhibitor cocktail was purchased from Roche Applied Science (Mannheim, Germany). Bicinchoninic Acid protein assay (BCA) was purchased from Thermo Fisher Scientific (Waltham, MA, USA), and PreOmics iST Sample Preparation Kit^®^ was purchased from PreOmics GmbH (Munich, Germany). Antibodies against carnitine palmitoyltransferase 1 (CPT1A, ab234111), peroxisome proliferator–activated receptor alpha (PPARα, ab24509), and total oxidative phosphorylation (OXPHOS, ab110413) rodent Western blot (WB) antibody cocktail were purchased from Abcam (Cambridge, MA, USA). Antibodies against voltage–dependent anionic channel (VDAC, GTX59911), alpha–smooth muscle actin (α–SMA, GTX10034), and CD36 (CD36, GTX55559) were purchased from Genetex (Irvine, CA, USA). Antibodies against NADH:ubiquinone oxidoreductase subunit A10 (NDUFA10, sc376357), ATP synthase F1 subunit beta (ATP5B, sc55597), phosphorylated adenine monophosphate protein kinase (pAMPK, sc109906), hypoxia–inducible factor 1α (HIF–1α, sc13515), and glyceraldehyde 3–phosphate dehydrogenase (GAPDH, sc25778) were purchased from Santa Cruz Biotechnology, Inc., (Dallas, TX, USA). IRDye^®^ secondary antibodies (680RD, 926–68074; 800RD, 926–32214; 680RD, 926–68073; 800RD, 926–32212; 800CW, 926–32213) were purchased from LI–COR Inc. (Lincoln, NE, USA). Tissue–Tek^®^ OCTMT was purchased from Finetek (Torrance, CA, USA).

### 2.2. Unilateral Ureteral Obstruction (UUO) Procedure

The Institutional Animal Care Committee (Comité Institucional para el Cuidado y Uso de Animales de Laboratorio, CICUAL) approved the experimental protocol at the “Facultad de Química de la Universidad Nacional Autónoma de México” (FQ/CICUAL/260/18). Animal handling and surgery procedures were conducted according to Mexican Official Norm Guides for the production, use, and care of laboratory animals (NOM–062–ZOO–1999) and the disposal of biological residues (NOM–087–SEMARNAT–SSA1–2002). Male Wistar rats (*n* = 28) with an initial body weight between 250 to 300 g were employed. For the surgical procedure, the rats were first anesthetized with isoflurane administered by inhalation; then, the abdominal area was shaved, and the antiseptic cleansing was performed; subsequently, a midline incision was made; and finally, lateral displacement of the intestines, the left kidney and ureter were identified. For the sham group, immediately after the ureter identification, the abdominal cavity was closed. For the UUO group, a double ligation of the left ureter below the kidney with a 3–0 silk suture was placed, and the abdominal cavity was closed. The animals were housed in a temperature–controlled environment with a 12:12 h light–dark cycle and maintained with water and food ad libitum. Euthanasia was performed using sodium pentobarbital (120 mg/kg, intraperitoneal). After this, kidneys were harvested in a cold buffer for the corresponding assays.

For proteomics, a total of 16 animals were employed, *n* = 4 per group, whereas for Western blots, 12 animals were employed, *n* = 3 per group.

### 2.3. Mitochondrial Isolation

Kidneys were rinsed and cooled by immersion in isolation buffer (225 mM D–mannitol, 75 mM sucrose, 1 mM EDTA, 5 mM HEPES, 0.1% BSA, pH = 7.4) at 4 °C. Renal cortex sections were obtained, cut into small pieces, and placed in 2 mL of cold isolation buffer, to be homogenized with a Potter Helvehjem tissue grinder. The homogenate was centrifugated at 800× *g* for 5 min at 4 °C; the supernatant was transferred to a pre–cooled tube and centrifuged 10,000× *g* for 15 min at 4 °C. The resulting pellet was gently disaggregated with 2 mL of albumin–free isolation buffer and centrifuged 10,000× *g* for 10 min at 4 °C. The mitochondria–containing pellet was resuspended in 150 µL of albumin–free isolation buffer and stored at −70 °C.

### 2.4. Sample Preparation for Mass Spectrometry

Mitochondrial pellets were lysed in lysis buffer (8 M Urea, 20 mM HEPES, 1 mM EDTA, pH 8.0) and sonicated for three cycles (10 sec at 4 °C per cycle) using a Sonic Dismembrator Model 100 (Fisher Scientific, Hampton, NH, USA), and were clarified by centrifugation at 16,000× *g* for 10 min; the protein content was quantified by bicinchoninic acid (BCA) assay. Then, 100 µg of mitochondrial extracts were precipitated using MeOH/Chloroform in 4:1 ratio. The resulting pellets were enzymatically digested using iST Sample Preparation Kit^®^ according to the protocol established by the manufacturer; briefly, 50 µL of “Lyse” reagent was added to protein pellets, placed in a heating block for 10 min, 95 °C with shaking, then, samples were sonicated for 20 cycles (30 s per cycle) using a BioRuptor Pico^®^ (Diagenode, Liège, Belgium). Protein samples were digested using 50 µL of a Lys–C/Trypsin mix (“Digest” reagent) and heating at 37 °C for 3 hrs. The resulting peptides were cleaned in an iST cartridge using “Wash 1” buffer to eliminate hydrophobic contaminants and “Wash 2” buffer to eliminate hydrophilic contaminants; afterward, peptides were eluted using “Elute” reagent and subsequently evaporated to dryness in a SpeedVac. Finally, peptides were resuspended with “LC–Load” reagent and stored at −80 °C until LC–MS analysis.

### 2.5. Label–Free Quantification by Mass Spectrometry and Data Analysis

Peptides were injected into the mass spectrometer Synapt G2–Si (Waters, Milford, MA, USA) in MS^E^ mode to calculate the area under the curve (AUC) of the total ion chromatogram (TIC), to normalize the injection prior to LC–MS analysis and, therefore, inject a comparable sample quantity for both conditions [23]. Afterwards, peptides in each sample were loaded and separated on an HSS T3 C18 column; 75 μm × 150 mm, 100 Å pore size, 1.8 μm particle size, using an UPLC ACQUITY M–Class with mobile phase A (0.1% formic acid in H_2_O) and mobile phase B (0.1% formic acid in acetonitrile) under the following gradient: 0 min 7% B, 121.49 min 40% B, 123.15 to 126.46 min 85% B, 129 to 130 min 7% B, at a flow of 400 nL·min^–1^ and 45 °C [23]. The spectra data were acquired in the mass spectrometer using nanoelectrospray ionization (nanoESI) and ion mobility separation (IMS) using the data–independent acquisition (DIA) approach through HDMS^E^ mode. The tune page parameters, for the ionization source, were set with the following values: 2.75 kV on the capillary emitter, 30 V on the sampling cone, 30 V on the source offset, 70 °C for the source temperature, 0.5 bar for the nanoflow gas and 150 L·h^−1^ for the purge gas flow. Two chromatograms were acquired (low and high energy chromatograms) in positive mode in a range of *m*/*z* 50–2000 with a scan time of 500 ms. No collision energy was applied to obtain the low energy chromatogram, while for the high energy chromatograms, the precursor ions were fragmented in the “transfer” using a collision energy ramp from 19 to 55 eV.

The MS and MS/MS measurements contained in the generated *.raw files were analyzed and relatively quantified using Progenesis QI for Proteomics software v4.2 (Waters, Milford, MA, USA) using a target decoy strategy against a *Rattus norvegicus* *.fasta database (obtained from UniProt, UP000002494, 29928 protein sequences, last modification on 30th May 2021), which was concatenated with the same *.fasta file in the reverse sense. Parameters used for the protein identification were trypsin as an enzyme and one missed cleavage allowed; carbamidomethyl (C) as a fixed modification and oxidation (M), amidation (C–terminal), deamidation (Q, N) and phosphorylation (S, T, Y) as variable modifications; default peptide and fragment tolerance (maximum normal distribution of 10 and 20 ppm respectively) and false discovery rate ≤4%. Synapt G2–S*i* was calibrated with [Glu1]–fibrinopeptide fragments through the precursor ion [M^+^ 2H]^2+^ = 785.84261 fragmentation of 32 eV with a result less than 2 ppm across all MS/MS measurements. The results generated from Progenesis software were exported to *.csv files to verify the figures of merit (FOM) described by Souza et al. for label–free experiments [24]: error histogram, peptide type classification, error distribution over mass range, separation by ion mobility of peptides, as well as protein dynamic range (Figures S1–S5); these plots were constructed using TIBCO Spotfire^®^ software v7.0.0 (TIBCO™, Palo Alto, CA, USA). Proteins considered differentially expressed display at least a ratio of ±1 (expressed as a base 2 logarithm); it means that these proteins had at least ± 2 absolute fold change, *p*-value ≤ 0.05, and two peptides (at least one unique peptide). The ratio was calculated based on the average MS signal response of the three most intense tryptic peptides (Top3) [25] of each characterized protein in all conditions by the Top3 of each protein in the SHAM sample.

### 2.6. Proteomics Statistical and Bioinformatics Analysis

The process used for the statistical analysis of the proteomic data was based on the methodology described previously [26] with some modifications. In the first part of our analysis, all identified and quantified proteins were checked for mitochondrial protein selection against a list of mitochondrial or mitochondrial traffic proteins reported by MitoMiner [27]. Subsequently, only those mitochondrial proteins with abundance values in at least 50% of the samples in their condition were selected. Then, missing values were imputed with the Random Forest method (missForest R package) [28].

Next, an exploratory analysis of the abundance data was performed with the Rapport package of R [29] to eliminate samples and proteins with extreme behavior from the analysis. Of the 16 samples (*n* = 4 per group), 12 samples were kept (*n* = 3 for sham, *n* = 3 for 7 days of UUO, *n* = 4 for 14 days of UUO and, *n* = 2 samples for 21 days of UUO); and of the 954 mitochondrial proteins (Table S1) selected with the MitoMiner database, 308 were chosen for the analysis.

Afterwards, a principal component analysis (PCA) was applied on the protein abundance correlation matrix [30] to obtain a mitochondrial protein abundance landscape for each UUO group and sham group.

Finally, only proteins with an absolute value of association equal to or greater than 0.5 with both first components [31] were selected for overrepresentation analysis of biological processes based on Gene Ontology (GO) [32]. This analysis was performed online employing the Gene List Analysis tool on the DAVID Bioinformatics Resources v6.8. As inputs, we uploaded the Entrez Gene ID as identifiers. An adjusted *p*-value < 0.05 was the cutoff criterion to identify significantly overrepresented biological processes [33,34].

### 2.7. Western Blot

For total protein extraction, 150–200 mg of renal cortex was disaggregated in 0.5 mL of radioimmunoprecipitation buffer (RIPA): 40 mM Tris–HCl, 150 mM NaCl, 2 mM EDTA, 1 mM EGTA, 5 mM NaF, 1 mM Na_3_VO_4_, 1 mM PMSF, 0.5% sodium deoxycholate, 0.1% SDS, pH 7.6 supplemented with protease inhibitor cocktail; using a Potter–Elvehjem homogenizer and centrifuged at 15,000× *g* for 10 min at 4 °C. Total protein in supernatants was quantified by BCA assay. Samples were prepared using 1:6 in Laemmli sample buffer (60 mM Tris–Cl, pH = 6.8, 2% SDS, 10% glycerol, 5% β–mercaptoethanol, 0.01% bromophenol blue). Equal amounts of proteins (25 µg) from each sample (*n* = 3 per group) were separated on 12% SDS–polyacrylamide gel electrophoresis (SDS–PAGE). Proteins were transferred to polyvinylidene fluoride (PVDF) membranes and blocked with 5% non–fat dry milk in tris–buffered saline plus 0.4% Tween–20 for 1 h at room temperature. Afterwards, membranes were incubated overnight at 4 °C with the appropriate primary antibody and the corresponding IRDye^®^ secondary antibody (1:10,000) for 2 h in darkness. The membranes were used for the immunodetection of multiple proteins. Protein bands were detected by the Odyssey Sa scanner (LI–COR Biosciences, Lincoln, NE, USA). Protein band density was analyzed with the Image Studio™ Lite Software LI–COR Odyssey (LI–COR Biosciences).

### 2.8. Statistical Analysis

The results obtained from the Western blot densitometry (*n* = 3 per group) were normalized against the loading control. The log10 of the intensity of the densitometry values was calculated to correct the variation between the technical replicates. Each technical replicate was standardized Z(µ = 0, σ) according to the following formula $z_{i}=\frac{x_{i}–\bar{x}}{ds}$.

The results obtained were analyzed with the R package Rapport [29] to eliminate outliers. The data were tested for normality and analyzed by one–way analysis of variance (ANOVA) for technical replicates, followed by Tukey’s multiple comparisons test. Data were plotted from three biological replicates per group, with one or two technical replicates, and every figure shows the mean with standard deviation (Prism 8.0 GraphPad Software, San Diego, CA, USA).

## 3. Results

### 3.1. Validation of Renal Fibrotic Damage and Mitochondrial VDAC Reduction during the UUO Progression

**Figure 1:**
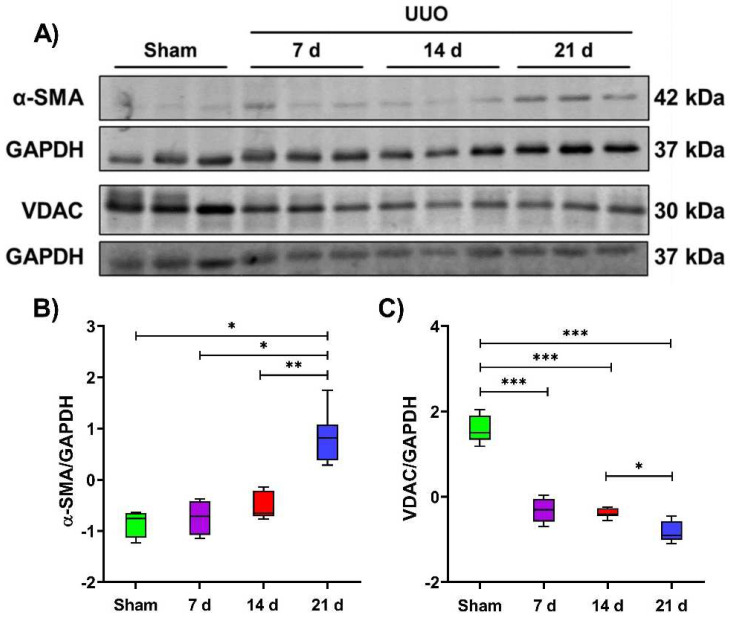
Mitochondrial VDAC alterations during the temporal course of unilateral ureteral obstruction (UUO). (**A**) Representative blots of the fibrotic marker alpha–smooth muscle actin (α–SMA) and the mitochondrial constitutive protein voltage–dependent anionic channel (VDAC) levels during 7, 14, and 21 days after UUO; glyceraldehyde 3–phosphate dehydrogenase (GAPDH) was used as a loading control. All Western blot measurements were performed in whole tissue lysate. (**B**,**C**) statistical analysis of the levels of α–SMA and VDAC. Data are represented as mean ± SD of two technical replicates from three rats per group; each technical replicate was standardized to Z(µ = 0, σ). * *p*  <  0.05, ** *p*  <  0.01, *** *p*  <  0.001.

To establish the progression of the UUO and how mitochondria are affected during this, we first assessed renal damage using alpha–smooth muscle actin (α–SMA) as an indicator of fibrosis development, observing, an increase during the time course of the UUO from 7 to 21 days after obstruction, as expected (Figure 1A,B). Subsequently, we assessed the level of the outer mitochondrial membrane protein voltage–dependent anion channel (VDAC), one of the most abundant constitutive proteins of mitochondria [35], evidencing a decrease from 7 to 21 days after obstruction (Figure 1C), which could suggest the loss of mitochondrial mass as previously reported [12], or even alterations in mitochondrial composition.

### 3.2. Kidney Mitochondrial Proteome Expression Profiles during UUO Progression

To explore changes in mitochondrial protein expression in obstructed kidney proteome along the time course of UUO and to analyze which metabolic pathways are involved, we first performed mitochondrial proteome identification by liquid chromatography coupled to tandem mass spectrometry (LC–MS/MS). We identified 954 proteins (Table S1), and after cross–checking with the MitoMiner database, we were left with 379 mitochondrial or mitochondrial transit proteins. Subsequently, only proteins that had abundance values in at least 50% of the samples per study group and did not show atypical abundance values were selected, thus conserving 308 mitochondrial proteins for subsequent analyses (Table S2).

**Figure 2:**
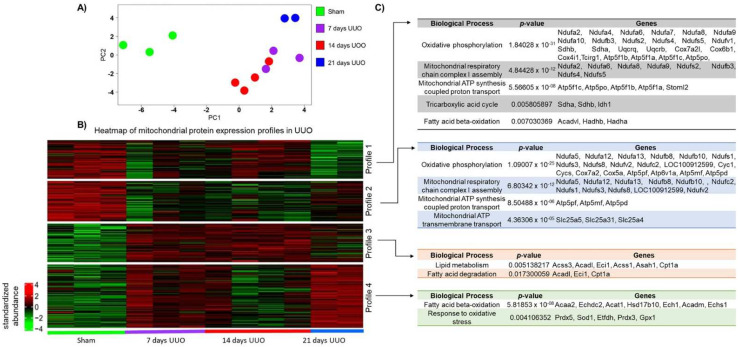
Analysis of the kidney mitochondrial proteome in a temporal course of the unilateral ureteral obstruction (UUO). (**A**) Principal component analysis of the kidney mitochondrial proteome in sham, 7, 14, and 21 days after UUO groups. (**B**) Heatmap of mitochondrial proteins abundance identified in sham, 7, 14, and 21 days of UUO groups. (**C**) Biological processes enrichment analysis performed for each of the profiles identified in the heatmap.

The PCA showed that the total variation in protein abundance among samples can be explained by 11 components (Table S3). The first component (PC1), which explains 41.63% of the total variation in the data, differentiates the sham group from the UUO groups. In addition, the second component (PC2), which explains 19.98% of the total variation in the data, demonstrated differences between the 21 days after the UUO group from those 7 and 14 days after UUO (Figure 2A).

Considering the results obtained from the PCA analysis, we constructed a heat map with 243 proteins that strongly correlated (r ≤ −0.5 or r ≥ 0.5) with the first two principal components (Table S4). The results showed the abundance patterns for each UUO day and for the sham group. Additionally, four expression profiles were distinguished. In profile 1, a set of proteins was found to be overexpressed in the sham group, moderately expressed at 7 and 14 days, and underexpressed at 21 days. In profile 2, similarly, the proteins of the sham group were overexpressed, whereas they were underexpressed from 7 to 21 days of the UUO. In profile 3, we observe the opposite effect with a set of underexpressed proteins in the sham group and overexpressed from 7 to 21 days after UUO. Finally, in profile 4, we observed a set of proteins with under and over–expression behavior during the progression of UUO, since in the sham group and at 14 days after UUO, we found underexpressed proteins, while at 7 and 21 days after UUO, we found an overexpression of the proteins (Figure 2B).

Subsequently, we performed an overrepresentation analysis of the biological processes with mitochondrial proteins of each of the four profiles described above (Table S5). We observed that the proteins that compose profile 1 are mainly involved in the OXPHOS (NDUFA2, NDUFA4, NDUFA6, NDUFA7, NDUFA8, NDUFA9, NDUFA10, NDUFB3, NDUFS2, NDUFS4, NDUFS5, NDUFV1, SDHA, SDHB, UQCRQ, UQCRB, COX7A2L, COX6B1, COX4I1, TCIRG1, ATP5F1B, ATP5F1A, ATP5F1C, and ATP5PO), complex I assembly (NDUFA2, NDUFA6, NDUFA8, NDUFA9, NDUFS2, NDUFB3, NDUFS4, and NDUFS5), ETS–coupled ATP synthesis (ATP5F1C, ATP5PO, ATP5F1B, ATP5F1A, AD STOML2), and TCA cycle (SDHA, SDHB, and IDH1) (Figure 2C). Similarly, for profile 2, we observed that proteins are mainly involved in OXPHOS (NDUFA5, NDUFA12, NDUFA13, NDUFAB8, NDUFB10, NDUFS1, NDUFS3, NDUFS8, NDUFV2, NDUFC2, LOC100912599, CYC1, CYCS, COX7A2, COX5A, ATP5PF, ATP6V1A, ATP5MF, and ATP5PD), complex I assembly (NDUFA5, NDUFA12, NDUFA13, NDUFB8, NDUFB10, NDUFC2, NDUFS1, NDUFS3, NDUFS8, LOC100912599, AND NDUFV2), ATP synthesis (ATP5PF, ATP5MF, and ATP5PD) and mitochondrial ATP transmembrane transport (SLC25A5, SLC2531, AND SLC25A4) (Figure 2C). Taken together, profiles 1 and 2 suggest disturbances in the OXPHOS and TCA cycle proteins during the UUO.

In the case of profile 3, the proteins found are mainly involved in lipid metabolism (ACSS3, ACADL, ECI1, ACSS1, ASAH1, and CPT1A) and fatty acid degradation (ACADL, ECI1, and CPT1A) (Figure 2C). Finally, for the proteins that compose profile 4, they are mainly involved in FA β–oxidation (ACCA2, ECHDC2, ACAT1, HSD17B10, ECH1, ACADM, and ECHS1) and in the oxidative stress response (PRDX5, SOD1, ETFDH, PRDX3, and GPX1) (Figure 2C).

In summary, the proteomic analysis demonstrates disturbance in proteins related to the OXPHOS, TCA, and lipid metabolism during the UUO progression.

### 3.3. OXPHOS Proteins Decrease in Renal Tissue during UUO Progression

**Figure 3:**
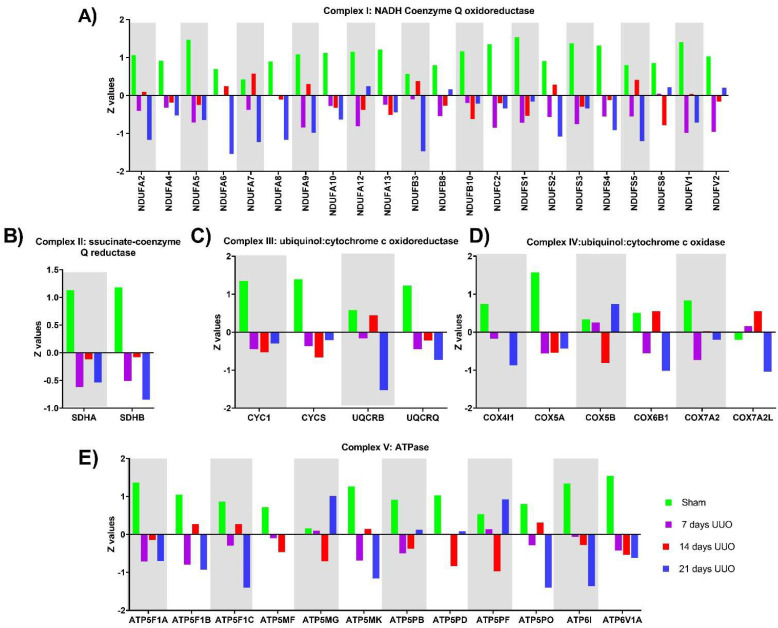
Differentially expressed proteins of electron transport system (ETS) proteins and ATP sybthase (ATPase) in kidney identified by proteomic during 7, 14, and 21 days after the unilateral ureteral obstruction (UUO). (**A**–**D**) The Z value of the abundance of subunits identified for complexes I, II, III, and IV of ETS in the kidney during UUO progression. (**E**) The Z value of the abundance of subunits identified for complexes V or ATPase in the kidney during UUO progression.

**Figure 4:**
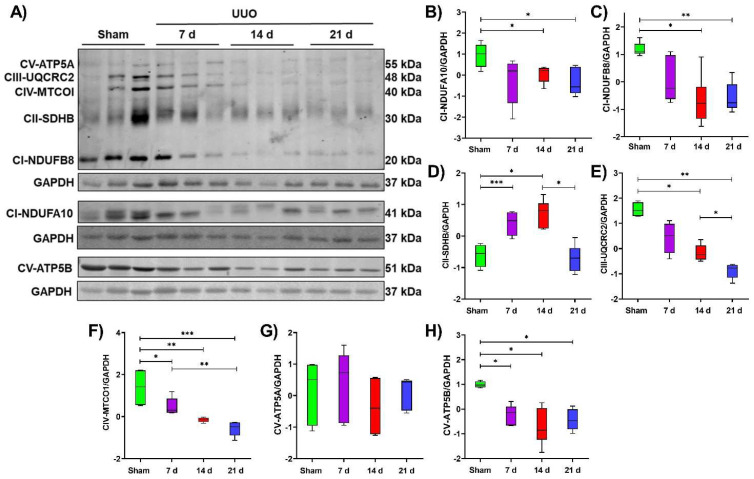
Representative OXPHOS protein levels in the temporal course of the unilateral ureteral obstruction (UUO). (**A**) Representative blots and (**B**–**H**) statistical analysis of the levels CI–NDUFB8, CI–NDUFA10, CII–SDHB, CIII–UQCRC2, CIV–MTCOI, CV–ATP5A, and CV–ATP5B subunits of the oxidative phosphorylation (OXPHOS) during 7, 14, and 21 days after UUO; glyceraldehyde 3–phosphate dehydrogenase (GAPDH) used as a loading control. All Western blot measurements were performed in whole tissue lysate. Data are represented as mean ± SD of two technical replicates from three rats per group; each technical replicate was standardized Z(µ = 0, σ). * *p*  <  0.05, ** *p*  <  0.01, *** *p*  <  0.001.

Since overrepresentation analysis of biological process from mitochondrial proteome profiles 1 and 2 revelated that OXPHOS proteins were among the most affected, we proceeded to reflect the expression of the subunits of each OXPHOS complex identified by proteomics of the enriched extract of mitochondria (Figure 3A–E) and also by Western blot in whole tissue lysate (Figure 4A).

Most complex I subunits identified by proteomics tended to decrease throughout the time course of UUO and markedly at 21 days. Although a decrease was observed at 7 and 21 days after UUO of NDUFA6, NDUFA7, NDUFA9, NDUFB3, NDUFS2, and NDUFS5 subunits, they reverted their behavior, increasing at 14 days of obstruction (Figure 3A).

Complex II is the smallest ETS complex, composed of only four subunits. In proteomic analysis, we identified a decrease in SDHA and SDHB subunits during the time course of the UUO compared to the sham group (Figure 3B).

As for complex I, most of the identified complex III subunits decreased during the UUO; nonetheless, the UQCRB subunit decreased at 7 and 21 days but increased at 14 days of the UUO with similar levels to the sham group (Figure 3C).

For complex IV, COX4I1, COX5A, and COX7A2 subunits were differentially decreased during UUO progression. Interestingly, the COX5B subunit decreased at 14 days after UUO, while it increased in the sham group, at 7 and 21 days after UUO. For COX7A2L and COX6B1 subunits, variations of under and overexpression were observed during UUO progression. For the COX7A2L subunits, decreased expression was observed in the sham group and at 21 days after the UUO, while increased expression was observed at 7 and 14 days after the UUO. In the case of the COX6B1 subunit, its expression was found to be increased in the sham group and at 14 days after the UUO, while at 7 and 21 days after the UUO it was found to be decreased. Moreover, at 21 days, all subunits were decreased, except for COX5B, which was found to increased (Figure 3D).

Finally, for ATP synthase, which is responsible for synthesizing ATP using the mitochondrial membrane potential, we observed decreased expression in most of the identified subunits in the different days of obstruction, except for the ATP5MG and ATP5PF subunits that show increased expression at 21 days of the UUO (Figure 3E).

Then, to have an overview of the OXPHOS system and to confirm the results obtained by proteomics in isolated mitochondria, we used in whole tissue lysate an antibody cocktail to detect the NDUFB8 subunit of complex I, SDHB subunit of complex II, UQCRC2 subunit of complex III, MTCOI subunit of complex IV, and the ATP5A subunit of the ATP synthase. In addition, the NDUFA10 subunit of complex I and ATP5B subunit of the ATP synthase also were evaluated separately.

Our results showed that NDUFB8 and NDUFA10 subunits of complex I tend to decrease at 7 days after UUO; and were significantly lower at 14 and 21 days of the UUO compared to the sham group (Figure 4B,C), supporting the observations of the proteomic analysis. Similarly, complex III subunit UQCRC2 tends to diminish at 7 days and decrease significantly at 14 and 21 days after obstruction compared to the sham group; moreover, a significant decrease was also observed at 21 days compared to 14 days of the UUO, suggesting that over the time course of the UUO, this complex III–subunit decreases progressively (Figure 4E). In the case of complex IV, the MTCOI subunit shows a significant decrease at 7, 14, and 21 days of the UUO compared to the sham group; in addition, a significant decrease was observed at 21 days compared to the 7 days UUO (Figure 4F). For ATP synthase, the subunits ATP5A show no differences, while the ATP5B subunit shows a significant reduction at 7, 14, and 21 after the UUO compared to the sham group (Figure 4H).

Interestingly, in the case of complex II, it showed a different trend to that found by proteomics since instead of observing a reduction in SDHB subunit levels, by Western blot, we observed a significant increase at 7 and 14 days after UUO compared to the sham group; and at 21 days after the UUO its levels were similar to those of the sham group (Figure 4D).

These results demonstrate that the components of OXPHOS tend to decrease during the progression of UUO, except for complex II.

### 3.4. Slight Alterations in Lipid Metabolism Proteins in Renal Tissue during the UUO Progression

**Figure 5:**
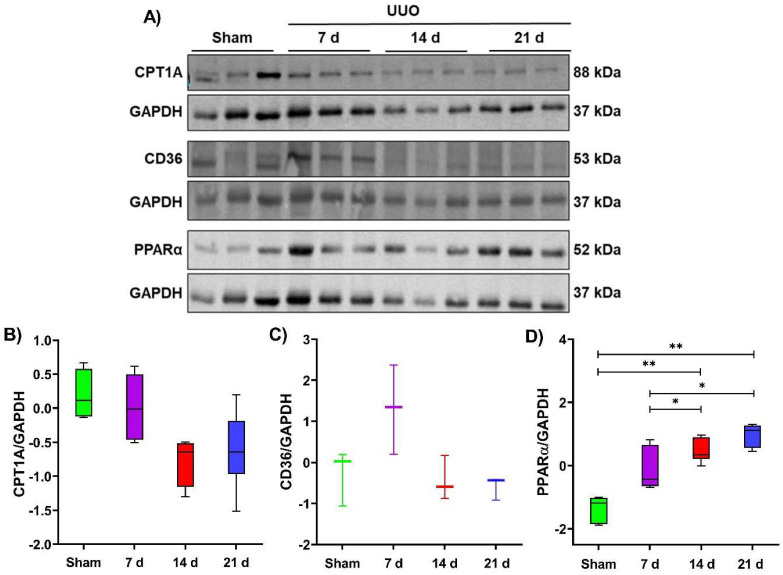
Fatty acid (FA) β–oxidation markers in the temporal model of the unilateral ureteral obstruction (UUO). (**A**) Representative blots of carnitine palmitoyltransferase 1 (CPT1A), CD36, peroxisome proliferator–activated receptor alpha (PPARα) as markers of fatty acid (FA) β–oxidation during 7, 14, and 21 days after the UUO, and glyceraldehyde 3–phosphate dehydrogenase (GAPDH) used as a loading control. (**B**–**D**) Expression of markers fatty acid (FA) β–oxidation. GAPDH was used as the loading control. All Western blot measurements were performed in whole tissue lysate. Data are represented as mean ± SD of one or two technical replicates from three rats per group; each technical replicate was standardized Z(µ = 0, σ). * *p*  <  0.05, ** *p*  <  0.01.

In addition to the OXPHOS and TCA cycle, the proteomic analysis of kidney mitochondria revealed lipid metabolism alterations during the UUO, observing that ACSS3, ACSS1, ACADL, and ECI1 are mainly overexpressed at 7 and 14 days after UUO (profile 3) and that ACAA2, ECHDC2, ACAT1, HSD17B10, ACADM, and ECHS1 proteins were overexpressed at 7 and 21 days after UUO (profile 4) (Figure 2C). As most of these proteins are involved in ketone bodies biosynthesis and FA β–oxidation, we proceeded to evaluate the levels of some representative proteins involved in lipid metabolism in whole tissue lysates. We evaluated the peroxisome proliferator–activated receptor alpha (PPARα), a key transcription factor in the induction of FA β–oxidation genes, and two relevant transporters for FA uptake present in the plasma membrane and mitochondria, the CD36 and carnitine palmitoyltransferase 1 (CPT1), respectively (Figure 5A).

Interestingly, PPARα increased during the progression of obstruction with significant increases at 14 and 21 days of the UUO compared to the sham group (Figure 4D); however, for the CD36 and CPT1 transporters, we found no significant differences between the sham and UUO groups. (Figure 5B,C). These results are interesting since PPARα is a transcription factor that regulates several genes involved in lipid transport and FA β–oxidation, including the plasma membrane and mitochondrial FA transporters CD36 and CPT1 [36]. Hence it seems that during the UUO, lipids could activate PPARα, but its transcriptional activity could vary since some products of its target genes, such as ACADL, ACAA2, and ACAT1, are increased; but not CD36 and CPT1. However, more studies are required to elucidate the dynamics of several proteins involved in lipid metabolism during the UUO.

### 3.5. Indirect Evaluation of Metabolic Reprogramming during the UUO Progression

**Figure 6:**
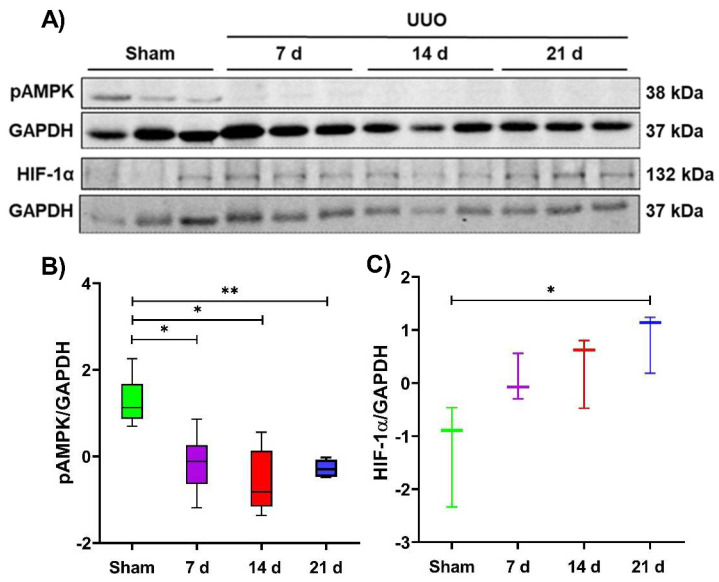
Metabolic reprogramming during the unilateral ureteral obstruction (UUO). (**A**) Representative blots of phosphorylated adenine monophosphate protein kinase (pAMPK) and hypoxia–inducible factor 1α (HIF–1α) during 7, 14, and 21 days after UUO; glyceraldehyde 3–phosphate dehydrogenase (GAPDH) was used as a loading control. (**B**) pAMPK and (**C**) HIF–1α statistical analysis. All Western blot measurements were performed in whole tissue lysate. Data are represented as mean ± SD of one or two technical replicates from three rats per group; each technical replicate was standardized Z(µ = 0, σ). * *p*  <  0.05, ** *p*  <  0.01.

FA–derived acetyl–CoA is the main substrate that fuels the TCA cycle and subsequent OXPHOS function in kidney mitochondria, especially in the tubular segment [11]; alterations in some of these metabolic processes, as occurs in the UUO, could drive to metabolic adaptations of renal cells trying to preserve their functionality and survive. Therefore, we evaluated the protein levels of two cellular energy sensors, the phosphorylated form of the adenine monophosphate–activated protein kinase (pAMPK) and the hypoxia–inducible factor 1α (HIF–1α) during the UUO progression (Figure 6A).

Our results showed that pAMPK levels decreased significantly on 7, 14, and 21 days of the UUO compared to the sham group (Figure 6B). Since pAMPK is induced by low energy status, its decrease suggests that during the UUO, ATP supply is enough and can be derived from other metabolic processes besides the OXPHOS, such as glycolysis.

Since HIF–1α is a transcriptional factor that promotes the expression of several genes involved in glycolysis [37,38], we next explored its levels in whole tissue lysates, observing that HIF–1α tends to increase along the UUO progression. However, a significant increase was observed only at 21 days of the UUO compared to the sham group (Figure 6C). These results suggest that metabolic reprogramming may occur in the UUO, as has been reported for kidney fibrosis development [20], since our results demonstrate that OXPHOS proteins are affected (Figure 2C), whereas HIF–1α increases as an indirect indicator of glycolysis promotion.

## 4. Discussion

The kidneys are organs with high–energy demand supplied principally by the mitochondria; thus, alterations in this organelle participate during the pathophysiology of different kidney diseases [39,40] as has been reported for the ON, in which changes in energy metabolism are associated with fibrosis development [12,20]. Since optimal energy metabolism is necessary for growth, proliferation, and other activities of the kidney cells, identification and understanding of mitochondrial metabolic alteration during ON are relevant and, in turn, would aid in the developing of new and effective therapies [38].

Previous reports from our group [12,41] and others [42] have shown that several histological changes occur during the progression of UUO, such as tubular dilatation, leukocyte infiltration, lipid deposition, and fibrosis development. In accordance with these changes, in this work, we indirectly confirm the fibrotic development by evaluating α–SMA. As we observed here, along with the progression of fibrosis, there is also mitochondrial dysfunction. In addition to the progression of fibrosis and renal damage that occurs during the progression of renal obstruction, concomitant alterations in mitochondrial structure and function have been reported [12], which led us to investigate how the mitochondrial proteome and its metabolic processes change during UUO.

Here, we demonstrated by a kidney mitochondrial proteome analysis the alterations in components belonging to OXPHOS, TCA cycle, and FA metabolism, mainly at 14 and 21 days of obstruction. In addition, we also observed changes in the energetic sensors pAMPK and HIF–1α in whole kidney tissue, which together suggest that metabolic reprogramming occurs during the UUO.

Our results show decreased levels of subunits of respiratory mitochondrial complexes, mainly of complex I and ATP synthase subunits (Figure 2C), demonstrated by kidney mitochondrial proteomics and Western blot analysis; these changes could be related to the OXPHOS dysfunction. Supporting this, a previous study from our group reported decreased respiratory parameters S3, S4o, and P in complex I–linked respiration from day 7 to day 28 of UUO, as well as the absence of changes in the respiratory control parameter (RC), indicating a decrease in mitochondrial–ATP production without uncoupling of ETS [12].

Regarding complex II, the proteomic analysis showed a decrease in the SDHA and SDHB subunits. However, SDHB levels measured by Western blot showed an increase at 7 and 14 days and returned to similar levels to the sham group at 21 days of the UUO (Figure 4B). These differences could be due to the fact that proteomic analysis was performed in isolated mitochondria samples; in contrast, Western blot analysis was performed on whole tissue lysate, suggesting that complex II subunits have not yet reached mitochondria and that their import to this organelle could be impaired. Complex II belongs to the OXPHOS system and also is part of the TCA cycle catalyzing the transformation of succinate to fumarate and, as a byproduct, producing the electron donor FADH_2_; therefore, in case of complex I dysfunction, as occurs in the UUO, complex II is able to feed the ETS to maintain OXPHOS. However, more in–depth studies are necessary for evaluating its function during the UUO since some findings also suggest that it could be affected at the first day of the UUO in mice due to the succinate accumulation in renal tissue [43].

Both proteomics and Western blot analysis revealed that certain subunits belonging to complexes III and IV decreased during UUO progression (Figure 3C,D, and Figure 4E,F). It is known that complex III is critical for ETS function for being an electron acceptor derived from complexes I and II through ubiquinone; hence, altered complex III functions are directly related to increased reactive oxygen species (ROS), which favor the progression of kidney diseases [44,45]. The proteomic analysis demonstrates that proteins involved in oxidative stress response also change during the UUO (Figure 2B,C); moreover, ROS have been reported to stabilize HIF–1α [46], thus probably contributing to metabolic reprogramming. Taken together, the reduced levels found in the subunits of complexes I, III, and IV reflect an impairment of the mitochondrial respiratory system, a condition that may underlie the reprogramming of energy metabolism in obstructed kidneys.

FA β–oxidation represents the main metabolic pathway that provides acetyl Co–A to fuel TCA and, subsequently, OXPHOS in the tubular segment of the kidney. Furthermore, through a proteomic approach, we revealed that proteins involved in the ketone body’s biosynthesis and FA β–oxidation are altered during the UUO (Figure 2C). Taking these results into account, we decided to evaluate in whole tissue lysate the levels of PPARα, a transcriptional factor that regulates the expression of several genes involved in FA metabolism [36], including some of the FA transport and β–oxidation. Our results also revealed increased levels from 7 to 21 days after UUO (Figure 5D). Although it has been reported that PPARa gene expression is decreased at 5 days after the UUO in mice [47], it could be that a rise in translation rate elicits increased protein levels, as our results demonstrate. The proteomic analysis demonstrates an increase in proteins involved in FA β–oxidation; however, CD36 and CPT1 levels in whole tissue lysate showed no differences in the UUO. This result could suggest that although FA β–oxidation is induced, FA uptake is limited, probably due to an excess of lipid deposition as observed during the UUO [12,48], other models of kidney fibrosis [49], and even in human kidney samples of chronic kidney damage (CKD) [50]. Strikingly, it has been reported that overstimulation of FA β–oxidation through the overexpression of CPT1 or the enhanced activation of PPARa with agonists in different kidney damage models can decrease fibrotic development [47,51]. Hence, an increase in PPARa and proteins involved in FA β–oxidation could be associated with an attempt to control or cope with the fibrosis progression.

In UUO, the decrease in pAMPK levels (Figure 6B) and increase in HIF–1α (Figure 6C) observed in our results suggest a metabolic reprogramming from OXPHOS metabolism to glycolysis, which might favor fibrosis development because previous reports have shown that inhibition of glycolysis reduces fibrotic progression in this model [19,20]. Likewise, previous studies have shown that HIF–1α expression increase during renal proximal tubule fibrosis leads to a reprogramming of cellular metabolism from FA β–oxidation to glycolysis and lipid accumulation [52]. In addition, the cellular adaptation to hypoxia could indicate that HIF1–α acts on several types of renal cells, such as myofibroblasts, to promote α–SMA expression [38].

Furthermore, a previous study reported that increased glycolysis can disrupt the TCA cycle [38]. Concerning this, in the proteomic analysis, we found that profile 1 reflected the presence of this metabolic process through the proteins SDHA, SDHB, and IDH1, which were found to be underexpressed during the UUO.

We consider it is essential to perform studies that help to understand how mitochondrial alterations contribute to ON pathophysiology. For this reason, we believe that the results obtained are relevant since, through a proteomic approach by LC–MS/MS, we show that alterations in mitochondrial bioenergetics such as OXPHOS, and the TCA cycle, and FA metabolism play an essential role in the progression of renal obstruction. Therefore, due to the critical role of mitochondrial alterations in the development of different nephropathies, mitochondria have been considered potential therapeutic targets in the treating of patients with kidney diseases. Based on the above, we consider our work’s perspective is useful in identification of proteins from the renal mitochondrial protein profiles which could be potential biomarkers of ON or therapeutic targets. In line with the latter, experimental results have shown that OXPHOS [53,54,55], inhibition of glycolysis [19,20], or enhancing FA metabolism [50,56,57,58,59] are potential therapeutic strategies related to the mitochondrial function that might decrease renal damage in the UUO.

**Figure 7:**
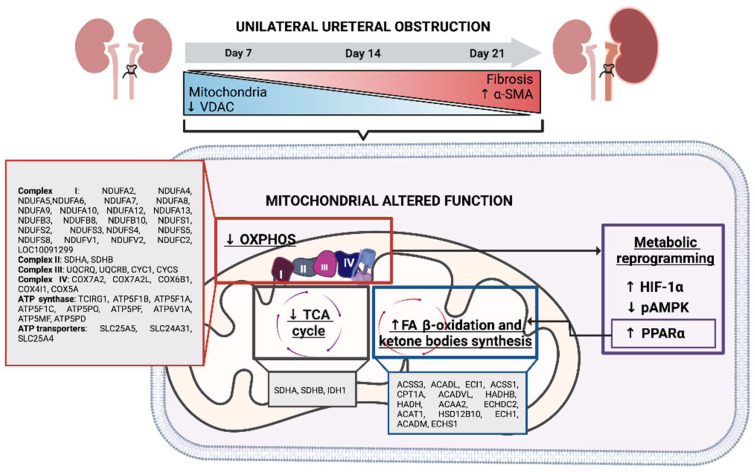
Integrative scheme. During the unilateral ureteral obstruction (UUO), mitochondrial VDAC decreases from 7 days after obstruction and continues during the obstruction. Mitochondrial dysfunction is attributed to the decrease of oxidative phosphorylation (OXPHOS), principally complex I (CI) and adenosine triphosphate (ATP) synthase subunits. In addition, the tricarboxylic acid (TCA) cycle is decreased, including the reduction of isocitrate dehydrogenase 1 (IDH1) and succinate dehydrogenase (SDH) enzymes. Interestingly, variations in β–oxidation are observed during the obstruction; however, an increase in the peroxisome proliferator–activated receptor alpha (PPAR α) is observed, suggesting an up–regulation of this process. All these mechanisms strongly contribute to mitochondrial dysfunction, which could induce the decrease of phosphorylated adenine monophosphate protein kinase (pAMPK) and the activation of hypoxia–inducible factor 1α (HIF–1 α), suggesting that metabolic reprogramming is triggered during obstruction. Thus, mitochondrial dysfunction and metabolic reprogramming might contribute to fibrosis development in the UUO.

In summary, this study demonstrated that changes in mitochondrial proteome expression during the UUO progression reflect alterations in OXPHOS, TCA, and FA metabolism associated with cellular adaptations to attempt to continue to meet the energetic demands of renal cells (Figure 7). Thus, it indicates that decreased mitochondrial function, but not uncoupling during UUO, drives a metabolic reprogramming that could contribute to the deterioration of renal function in the UUO. We also demonstrate that through a high–throughput tool such as proteomics, accompanied by an appropriate statistical analysis such as PCA, we can identify those mitochondrial proteins that are primarily involved in UUO progression.

## 5. Conclusions

In this study, we focus on changes in kidney mitochondrial proteome during the progression of UUO that reveal significant alterations in the OXPHOS, TCA, and FA metabolism of mitochondrial energy metabolism pathways leading to metabolic reprogramming of renal cells, which in addition to attempting to maintain cellular function, may also contribute to the development of fibrosis. Furthermore, although a reduction of most subunits of the ETS and ATP synthase was observed during UUO, overrepresentation analysis of biological processes in mitochondrial proteome of the obstructed kidney suggests that complex I and ATP synthase are the most altered during the UUO.

Finally, from the perspective of this work, it would be interesting to evaluate the renal mitochondrial proteome profiles proposed here to identify proteins as potential biomarkers for ON and their potential use as therapeutic targets.

## Data Availability

The mass spectrometry proteomics data have been deposited at the ProteomeXchange Consortium via the PRIDE [60] partner repository with the dataset identifier PXD036205.

## Supplementary Materials

The following supporting information can be downloaded at: https://www.mdpi.com/article/10.3390/metabo12100936/s1, Figures S1–S5, and Tables S1–S5.
