# Supplementary material for: Expression Profiles of Kidney Mitochondrial Proteome during the Progression of the Unilateral Ureteral Obstruction: Focus on Energy Metabolism Adaptions
Source: Metabolites. 2022 Oct 2;12(10):936. doi: 10.3390/metabo12100936 (PMC9607257; doi:10.3390/metabo12100936)
Supplement: Supplementary file 1 [file metabolites-12-00936-s001.zip › metabolites-1903399-Supplementary_Figures.pdf]

metabolites

MDPI

Supplementary Material

Expression Profiles of Kidney Mitochondrial Proteome during the Progression of the Unilateral Ureteral Obstruction: Focus on Energy Metabolism Adaptions

Ariadna Ortega-Lozano [1,†], Alexis Paulina Jiménez-Uribe [1,†], Ana Karina Aranda-Rivera [1], Leopoldo Gómez-Caudillo [1], Emmanuel Ríos-Castro[3], Edilia Tapia [2], Belen Bellido [1], Omar Emiliano Aparicio-Trejo [2], Laura Gabriela Sánchez-Lozada[2], and José Pedraza-Chaverri [1,*]

1 Department of Biology, Faculty of Chemistry, National Autonomous University of Mexico (UNAM), Mexico City 04510, Mexico
2 Unidad de Genómica, Proteómica y Metabolómica (UGPM), LaNSE, Cinvestav-IPN, Mexico City 07360, México.
3 Department of Cardio-Renal Physiopathology, National Institute of Cardiology "Ignacio Chávez", Mexico City 14080, Mexico
* Correspondence: pedraza@unam.mx; Tel./Fax: +52-55-5622-3878
† These authors contributed equally to this work.

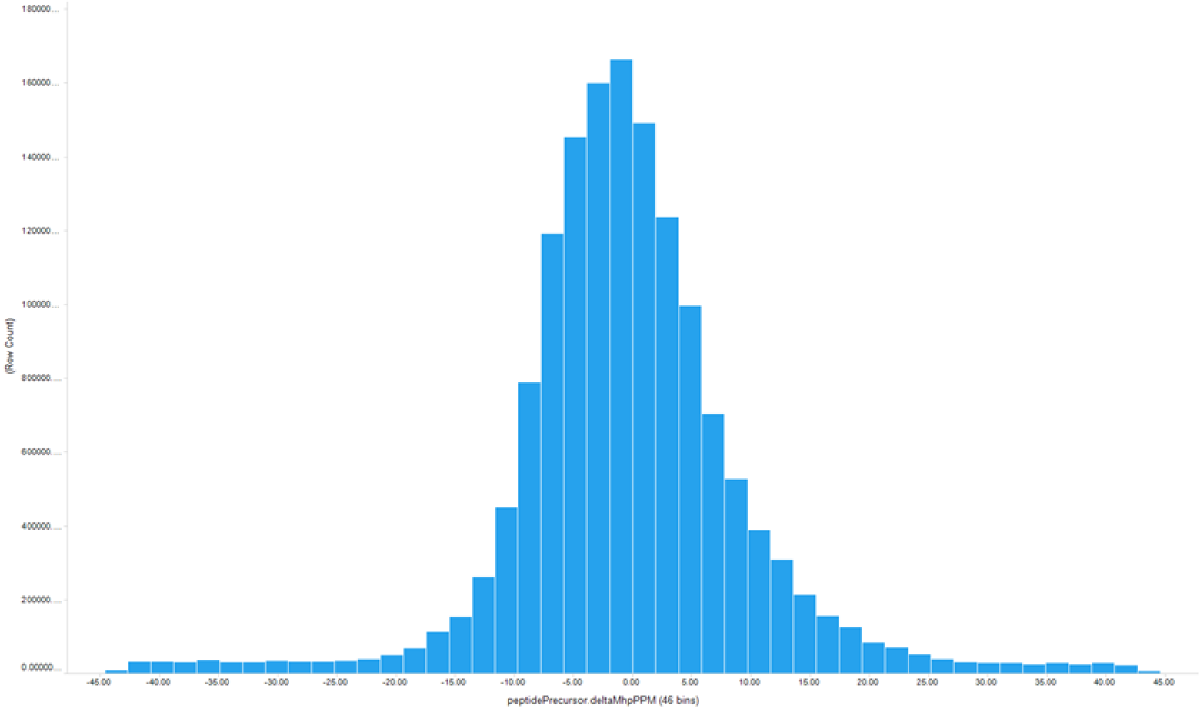

Figure S1. Histogram of detected peptides. Histogram representing a total of 192,196 detected peptides; 84.19% of them fall into an error maximum of ± 10 ppm, indicating that the calibration of the mass spectrometer was adequate.

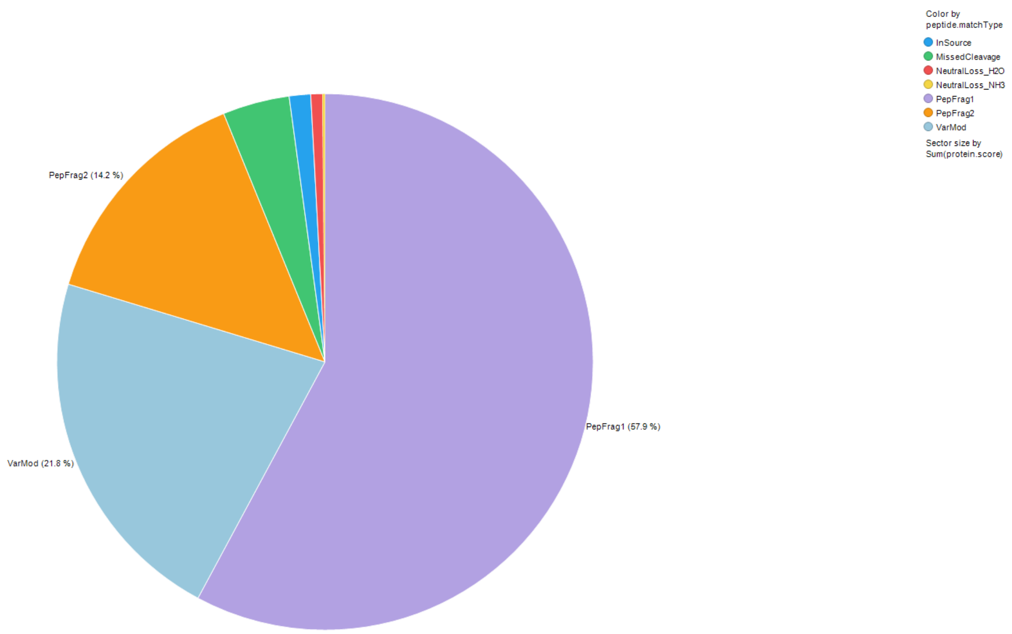

Figure S2. Pie chart representing the types of peptides identified, most of them (79.7%) are peptides of high quality denominated PepFrag1 (57.9%) and peptides with some modifications (including post-translational modifications) called Varmod (21.8%); 14.2% were considered as PepFrag2 which represent peptides with less reliability because they were identified with less restrictions during database search, and finally, missed cleavages peptides (green), neutral loss of H_2O (red) and NH_3 (yellow) as well as fragmented peptides at ion source (blue) represent <6.1% of total peptides, which is desirable in proteomic experiments.

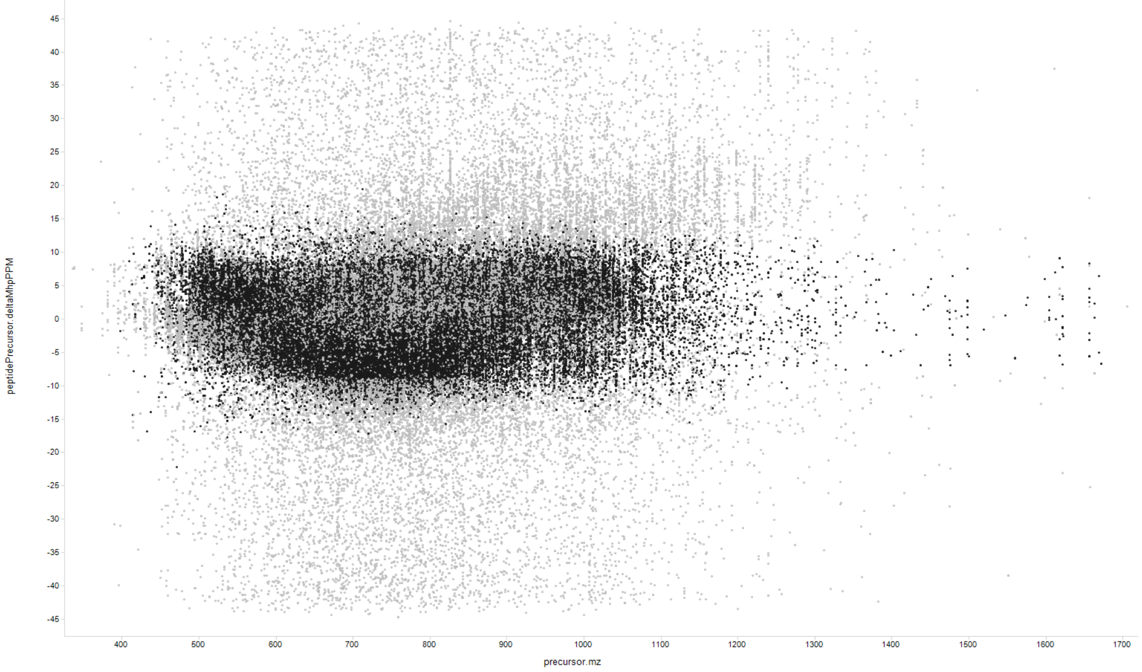

Figure S3. Error vs m/z range plot. Most of high-quality peptides are concentrated at a maximum of ± 10 ppm throughout the analyzed m / z range (black dots), besides these peptides are condensed mostly in m/z 500-1000, which is expected in proteomic experiments.

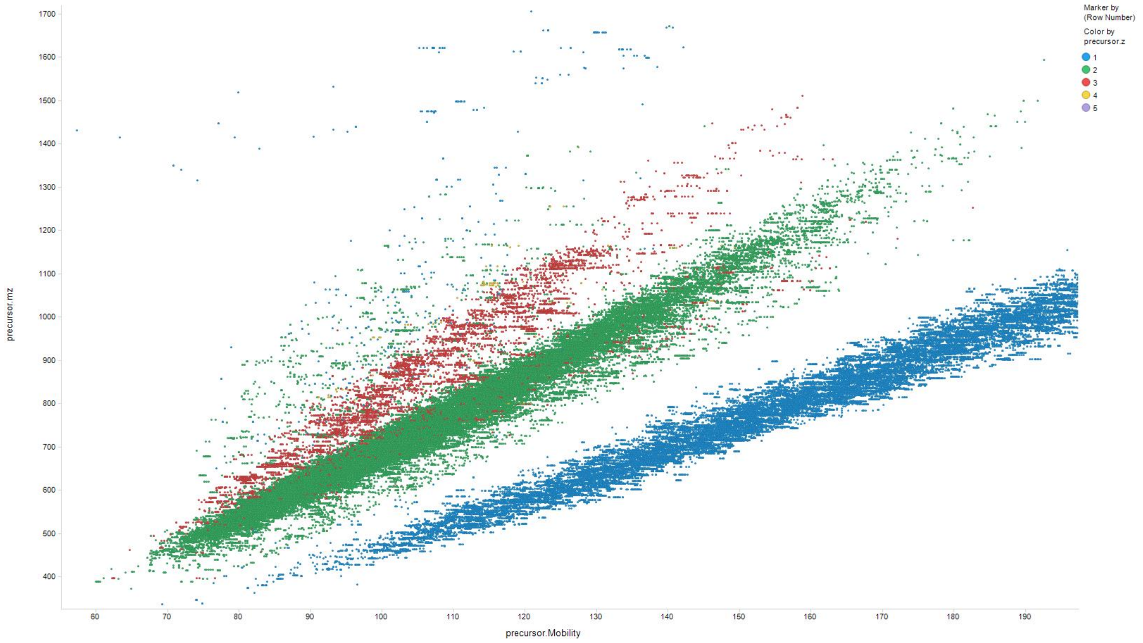

Figure S4. Ion mobility vs m/z plot. Plot which represents the way in which ions are moving inside of the mobility cell of *Synapt G*2-*Si* based on their Drift Time. Clusters of ions with different charge states were observed indicating a correct separation influenced by IMS. Ions with z=1^{+} (blue dots), ions with z=2^{+} (green dots), ions with z=3^{+} (red dots), other colors are ions with superior charge states. .

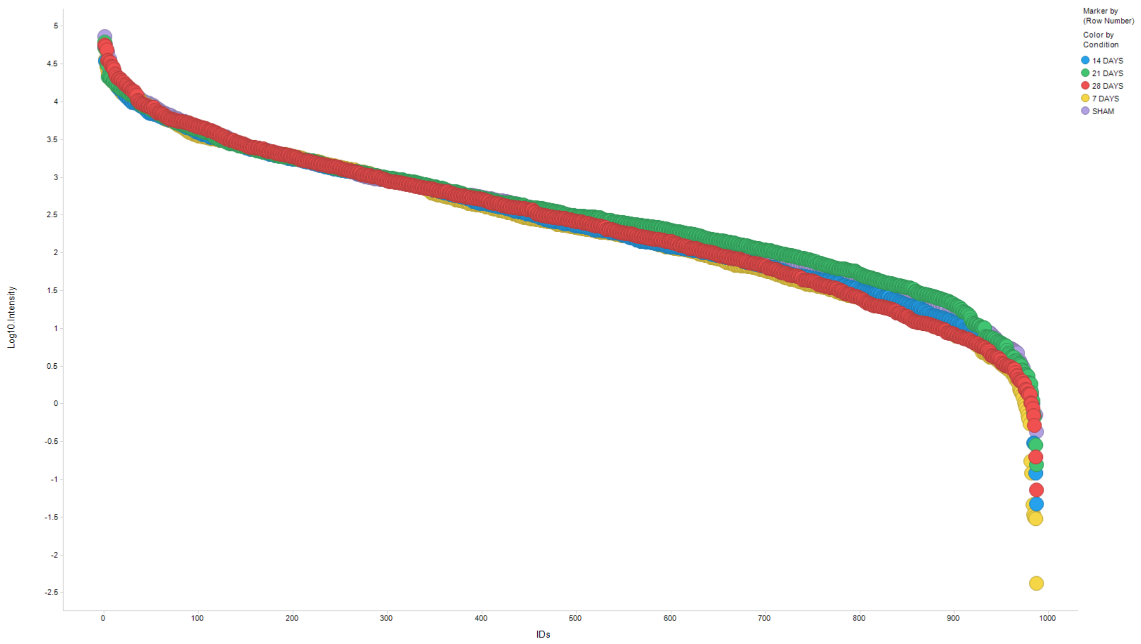

Figure S5. Dynamic range of quantified proteins. In purple, yellow, blue, green, and red dots, correspond to proteins from SHAM, 7OUU, 14OUU, 21OUU, 28OUU samples respectively. Proteins were expressed in ≈ 6.5 magnitude orders and, the behavior of the dynamic range of the proteins in all conditions was similar, so the normalization of the injection was adequate. Abscissa axis corresponds to the number quantified proteins (ID´s); ordinate axis corresponds to the average of Top3 intensities for each detected protein (values are represented as base 10 logarithm).
